# Immune Landscape of Gastric Carcinoma Tumor Microenvironment Identifies a Peritoneal Relapse Relevant Immune Signature

**DOI:** 10.3389/fimmu.2021.651033

**Published:** 2021-05-13

**Authors:** Chuang Zhang, Danni Li, Ruoxi Yu, Ce Li, Yujia Song, Xi Chen, Yibo Fan, Yunpeng Liu, Xiujuan Qu

**Affiliations:** ^1^ Department of Medical Oncology, The First Hospital of China Medical University, Shenyang, China; ^2^ Key Laboratory of Anticancer Drugs and Biotherapy of Liaoning Province, The First Hospital of China Medical University, Shenyang, China; ^3^ Liaoning Province Clinical Research Center for Cancer, The First Hospital of China Medical University, Shenyang, China; ^4^ Key Laboratory of Precision Diagnosis and Treatment of Gastrointestinal Tumors, Ministry of Education, The First Hospital of China Medical University, Shenyang, China; ^5^ Department of Radiation Oncology, Cancer Hospital of China Medical University, Liaoning Cancer Hospital & Institution, Shenyang, China

**Keywords:** gastric cancer, LASSO, peritoneal relapse, TME, immune signature

## Abstract

**Background:**

Gastric cancer (GC) still represents the third leading cause of cancer-related death worldwide. Peritoneal relapse (PR) is the most frequent metastasis occurring among patients with advanced gastric cancer. Increasingly more evidence have clarified the tumor immune microenvironment (TIME) may predict survival and have clinical significance in GC. However, tumor-transcriptomics based immune signatures derived from immune profiling have not been established for predicting the peritoneal recurrence of the advanced GC.

**Methods:**

In this study, we depict the immune landscape of GC by using transcriptome profiling and clinical characteristics retrieved from GSE62254 of Gene Expression Omnibus (GEO). Immune cell infiltration score was evaluated *via* single-sample gene set enrichment (ssGSEA) analysis algorithm. The least absolute shrinkage and selection operator (LASSO) Cox regression algorithm was used to select the valuable immune cells and construct the final model for the prediction of PR. The receiver operating characteristic (ROC) curve and the Kaplan-Meier curve were used to check the accuracy of PRIs. Gene Set Enrichment Analysis (GSEA) and Kyoto Encyclopedia of Genes and Genomes (KEGG) analysis were performed to explore the molecular pathways associated with PRIs.

**Results:**

A peritoneal recurrence related immune score (PRIs) with 10 immune cells was constructed. Compared to the low-PRIs group, the high-PRIs group had a greater risk. The upregulation of the focal adhesion signaling was observed in the high-PRIs subtype by GSEA and KEGG. Multivariate analysis found that both in the internal training cohort and the internal validation cohort, PRIs was a stable and independent predictor for PR. A nomogram that integrated clinicopathological features and PRIs to predict peritoneal relapse was constructed. Subgroup analysis indicated that the PRIs could obviously distinguish peritoneal recurrence in different molecular subtypes, pathological stages and Lauren subtypes, in which PRIs of Epithelial-Mesenchymal Transitions (EMT) subtype, III-IV stage and diffuse subtype are higher respectively.

**Conclusion:**

Overall, we performed a comprehensive evaluation of the immune landscape of GC and constructed a predictive PR model based on the immune cell infiltration. The PRIs represents novel promising feature of predicting peritoneal recurrence of GC and sheds light on the improvement of the personalized management of GC patients after surgery.

## Introduction

Gastric cancer (GC) ranks the fifth in prevalent lethal malignancies and the third in cancer-related death worldwide (1). Of the patients with advanced-stage GC, most of them develop liver, lymph nodes and peritoneum metastasis within 5 years after radical surgery. Among these metastases, peritoneal dissemination is the most frequent and lethal, especially in the serosa-invasive gastric cancers (2). Although the conventional clinicopathological detections such as medical imaging and cytological examination of peritoneal effusion have been applied to assess the relapse, the prediction of PR is not accurate and sensitivity enough (3). Despite optimal treatment including corrective surgery (CRS), systemic chemotherapy, intraperitoneal chemotherapy and hyperthermia intraperitoneal chemotherapy (HIPEC), they are not adopted as the first-line strategy due to the controversial outcomes after long-term follow-up (4–6). In summary, it is urgently needed to construct an individual approach or model for predicting the PR risk of GC.

The tumor immune microenvironment (TIME) is the environment of malignant tumor progression, in which the host antitumor immune response and normal tissue destruction occur (7–12). Accumulating evidence indicated the key role of immune cells infiltration in the peritoneal metastasis of different cancers including GC (13–16). It is observed that GC patients with peritoneal metastasis had increased levels of alternatively activated macrophages in the peritoneum compared to those without dissemination. The underlying mechanism is that macrophages in the peritoneum of GC patients play a supportive role in peritoneal metastasis by producing EGF and VEGF (17). Besides, Rihito Kanamaru reported that neutrophil extracellular traps (NETs) on peritoneal surface can promote the clustering and growth of free tumor cells disseminated in abdomen, which was assisted by low-density neutrophils in postoperative abdominal cavity. Although several studies have explored the relationship between immune infiltration and prognosis of GC, to date, the probable correlation between peritoneal metastasis and the landscape of immune cells infiltrating has not yet been exclusive (18–20).

With the goal of improving precise prediction of PR after curative surgery of gastric cancer, in the current study, we applied the algorithm ssGSEA, which has been deemed to be the most accurate method available (21). ssGSEA is a newly proposed computational algorithm for enumeration of immune cell subsets using RNA specimens from multiple tissue types, including solid tumors, and has outperformed other methods regarding noise, unknown mixture content and closely related cell types. This study aims to construct a novel prediction system specific to PR, showing the immune infiltration landscape of patients with or without peritoneal relapse after surgery. As a result, we established a methodology to quantify the PR related immune score (PRIs) with an integrated analysis of the infiltration status of 24 immune cells, which was found to be a robust predictor of PR. Therefore, the PRIs represents novel promising signature for predicting peritoneal recurrence of GC after surgery.

## Material and Methods

### Search and Collection of Cohort With the Pathological Data of Peritoneal Relapse

To identify gastric cancer gene expression profile data with clinical pathological data of PR, a systematic search was performed on the Gene Expression Omnibus (GEO) data set (https://www.ncbi.nlm.nih.gov/geo/). The search strategy is (((“peritoneum”[MeSH Terms] OR peritoneal[All Fields]) AND (“neoplasm metastasis”[MeSH Terms] OR metastasis[All Fields])) OR ((“peritoneum”[MeSH Terms] OR peritoneal[All Fields]) AND (“recurrence”[MeSH Terms] OR relapse[All Fields])) OR ((“peritoneum”[MeSH Terms] OR peritoneal[All Fields]) AND (“recurrence”[MeSH Terms] OR recurrence[All Fields]))) AND (“stomach neoplasms”[MeSH Terms] OR (((((((gastric cancer[Title] OR gastric adenocarcinoma[Title]) OR gastric tumor[Title]) OR gastric carcinoma[Title]) OR stomach cancer[Title]) OR stomach adenocarcinoma[Title]) OR stomach tumor[Title]) OR stomach carcinoma[Title])). A total of 45 items were obtained from the initial screening. After further manual identification, and under the premise that the number of cases is not less than 100, Finally, the two cohorts GSE62254 and GSE10581 passed the review and their corresponding raw data were downloaded. All 300 samples of GSE62254 and 108 samples of GSE15081 with clear clinical parameters of peritoneal relapse were selected for further analysis. The corresponding clinicopathological parameters are summarized in **Table S1**.

### Analysis of Immune Cell Infiltration

The Single sample gene set enrichment analysis (ssGSEA) method is a further extension of the GSEA method (21), it can define the absolute enrichment score of a certain immune cell marker genes dataset in a particular patient. We used ssGSEA to calculate the immune infiltration score for each immune cell in each patient and normalized enrichment scores were used for subsequent analysis. The markers genes of immune cells were obtained from the work of Jérôme Galon et al. (22) (**Table S2**). The above is achieved by the R package GSVA (23).

### Establishment of the Lasso-Cox Model

The process of establishing and verifying the PRIs model is displayed in **Supplement Figure 1A**. Firstly, a total of 300 patients of GSE62254 cohort included in the study were randomly assigned to a 1:1 training set and validation set. Secondly, the hazard ratio of PR of immune cells in the training set was calculated using the univariate cox proportional hazard regression model. Then Immune cells that were meaningful for univariate analysis in the training set are included in the penalized Cox regression model with least absolute shrinkage and selection operator (LASSO) Cox regression model for ten-fold cross validations to select the most significant immune cells for PR (24). Finally, a PR-related immune model was constructed based on the immune cells.

### ROC Curve

Receiver operating characteristic curve (ROC), area under the curve (AUC) and calibration curve were obtained using R packages “pROC” and “rms”.

### KEGG Analysis Based on PRIs

Kyoto Gene and Genomic Encyclopedia (KEGG) analysis was used to determine the biological pathways in which genes associated with PRIs were significantly enriched. Pathways with adv. less than 0.5 were considered meaningful. The above is achieved by the R package clusterProfiler (25).

### Gene Set Enrichment Analysis (GSEA)

GSEA v2.2.2 (http://www.broadinstitute.org/gsea) was used to investigate the biological difference between patients with high or low PRIs. C2:CP KEGG gene sets from MSigDB were used as the reference gene sets. All other parameters were set to default.

### Construction and Validation of PRIs Related Nomogram

The PRIs and pStage with the stable value of predicting PR were used to construct the final nomogram. R package nomogramEx was used to calculate and plot. DCA was used to identify the clinical benefit of PRI-related nomogram.

### Statistical Analysis

Continuous variables were analyzed using Student’s t-tests or nonparametric tests. Categorical variables were analyzed using Chi-squared tests or Fisher’s exact tests. R package survival and survminer was used for survival analysis. R package coxph was used for univariate and multivariate analyses. The ROC curve was plotted by R package survivalROC. All data were analyzed by SPSS and R software (http://www.r-project.org/). The results with P < 0.05 were considered statistically significant.

## Results

### Overview of Differential Patients’ Outcomes Between PR+ and PR- Patients in GSE62254 Cohort

**Figure 1:**
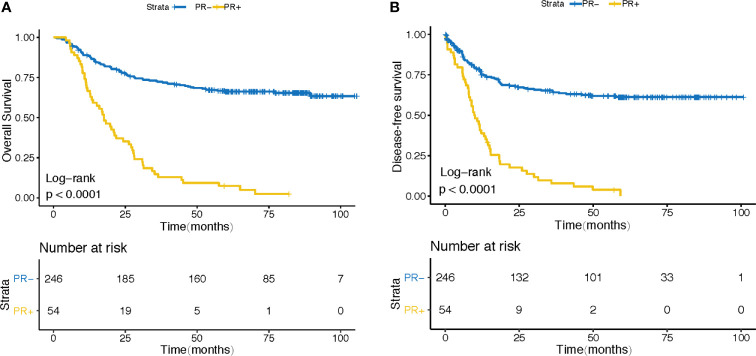
Association between PR status and patients’ outcomes. **(A)** for OS and **(B)** for PFS.

In order to clarify the effect of PR on the overall survival (OS) and progression free survival (PFS) of gastric cancer patients after surgery, we verified it in GSE62254 cohort. 300 patients were divided into PR- and PR+ groups according to their PR status after surgery. As expected, patients in PR+ subgroup demonstrated a worse prognosis (**Figure 1A**) and a higher recurrence rate after surgery (**Figure 1B**).

### Immune Landscape and the TME Characteristics in GSE62254 Cohort

**Figure 2:**
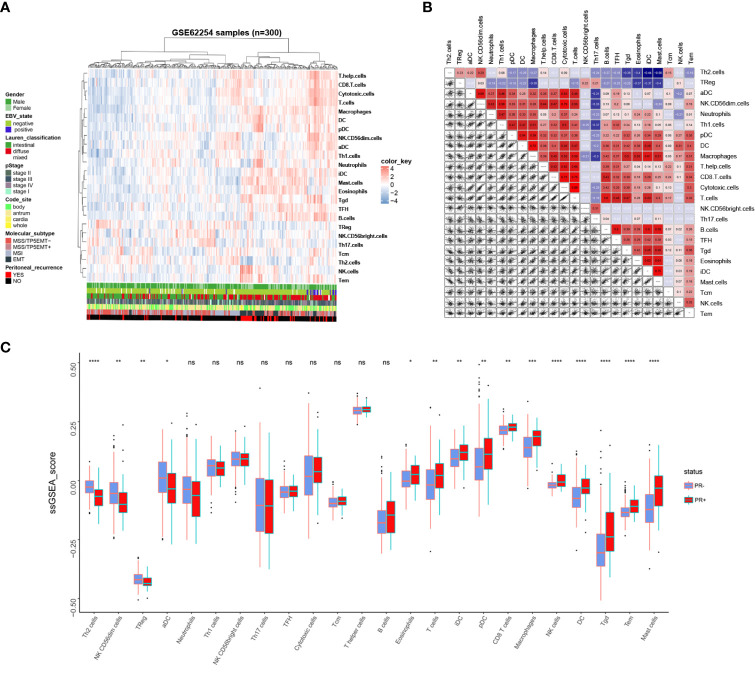
Immune landscape of GC and the TME characteristics. **(A)** Unsupervised clustering of GC patients from the GSE62254 using ssGSEA score calculated from immune cells. **(B)** Correlation of the TME immune cells. **(C)** The Relative immune infiltration score of 24 immune cells between PR- and PR+ gastric tissues. *P < 0.05, **P < 0.01, ***P < 0.001, ****P < 0.0001, ns: no significance.

Immune cells infiltration status was calculated using R package “GSVA”. A total of 24 immune-related cells were included to evaluate the immune infiltration status of tumor tissues. Immune infiltration landscape and corresponding clinical parameters are shown in **Figure 2A**. As can be seen from the heatmap, patients with PR+ conducted different immune infiltration status from patients with PR-, and the infiltration abundance of different immune cells in the same patient is different. To better understand the relationship between various immune cells in gastric cancer tissues, we constructed a correlation analysis among the 24 immune cells (**Figure 2B**). The results showed that T cells, cytotoxic cells, helper T cells and CD8+T cells were highly correlated. There is also a high correlation between Th2 cells and Treg cells. Besides, Mast cells, eosinophils and IDC cells were highly correlated. Moreover, there is a high correlation between NK cells and Tem cells. Therefore, this suggests that they may be involved in the same biological behavior in the immune microenvironment of gastric cancer that promotes peritoneal recurrence of gastric cancer. In order to verify whether the immune infiltration status of patients with different PR status is different, we divided 24 immune cells into PR+ and PR- groups according to the PR status. As **Figure 2C** showed, 15 out of 24 immune cells presented different immune infiltration status between PR+ and PR- groups. Consistent with the results of **Figure 2B**, NK cell and Tem were consistently highly infiltration in PR+ patients, PR+ patients also possessed consistently lower Th2 and TReg cells infiltration, and consistently higher infiltration of mast cell, eosinophils and iDC. Then we verified the infiltration scores of 24 types of immune cells in PR+ and PR- groups in GSE15081, and the results were similarly (**Supplement Figure 1B**). This makes us realize that immune cells may play an important role in PR, and the infiltration of immune cells can reflect the PR of patients.

### Feature Selection and Construction of PRIs Signature

**Figure 3:**
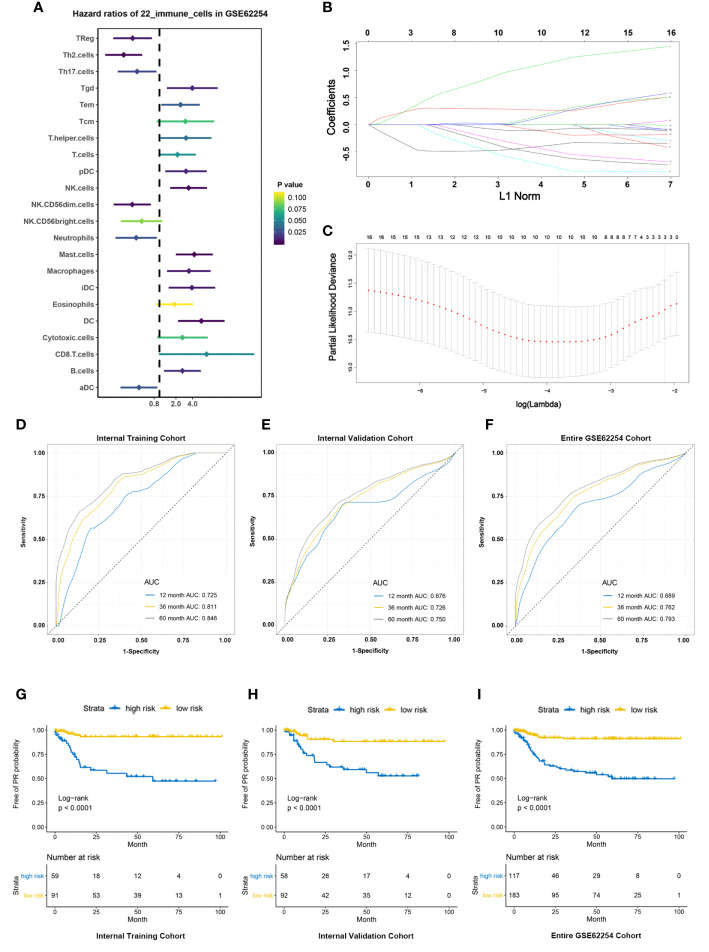
Construction and validation of PRIs signature. **(A)** Forest plots showing associations between different immune cells and PR risk in the training cohort. **(B)** LASSO coefficient profiles of the fractions of immune cells. **(C)** Parameter selection for tuning by 10-fold cross validation in the LASSO model. **(D–F)**. PRIs measured by time-dependent receiver–operating characteristic (ROC) curves in the training cohort, validation cohort, entire cohort at 1, 3 and 5 years respectively. **(G–I)** KM-curve for patients with high and low PRIs in the training cohort, validation cohort, entire cohort respectively.

First of all, all 300 patients in GSE62254 cohort were randomly divided into training cohort and validation cohort according to the ratio of 1 to 1. The survminer package in R software was used to calculate the optimal cutoff value for each immune cell in the training cohort (**Table S3**). Based on the cutoff, the Infiltration fraction score of each immune cell was valued as 0 or 1. Next, univariable analysis of the immune cells in the training cohort was performed to calculate the association between each immune cell and the risk of PR (**Figure 3A**). Finally, ten-fold cross validation LASSO Cox regression analysis was used to build a PR risk evaluation model (PRIs) in the training cohort using the immune cells associated with peritoneal recurrence (**Figures 3B, C**) and we constructed an PRIs formula:

$$\begin{array}{c} \text{PRIs }=\;-(0.405\;\times \text{ fraction level of Th}2\text{ cells})\;+\;(0.270\times \text{ fraction level of Mast cells})\\ +\;(1.093\;\times \text{ fraction level of DCs})-\;(0.712\;\times \text{ fraction level of NK}.\text{CD}56\text{dim}.\text{cells})\\ \;-\;(0.527\times \text{ fraction level of Tregs})\;+\;(0.155\;\times \text{ fraction level of Tgd})\\ -\;(0.429\;\times \text{ fraction level of Th}17\text{ cells})\;-\;(0.103\times \text{ fraction level of Neutrophils})\\ -\;(0.107\times \text{ fraction level of aDC }\mathrm{cells})\;+\;(0.121\times \text{ fraction level of T helper cells}) \end{array}$$

In the training cohort, the accuracy of PRIs was investigated at time points 2, 3, and 5 years and the AUC is 0.725,0.811,0.846 respectively (**Figure 3D**), while in the validation cohort is 0.676,0.726,0.750 respectively (**Figure 3E**). In all 300 patients, the AUC of PRIs at time points 2, 3, and 5 years is 0.689,0.762,0.793 respectively (**Figure 3F**). In addition, we further applied the obtained PRIs score formula to the external validation cohort, due to the limitation of the external validation cohort itself, we can only get the PR status at time point 3 years. ROC at time point 3 was 0.690, which shows that the PRIs has a significant stability (**Supplement Figure 1C**). According to the cutoff value (-0.508) obtained through the survminer package, the patients in the training cohort were divided into high and low PRIs groups. Patients with high PRIs accumulate more PR events and had a worse prognosis (**Figure 3G**). When applying the cutoff value to the validation cohort and all patients in GSE62254, the conclusion is consistent (**Figures 3H, I**).

### Association Between PRIs and Clinicopathological Parameters

**Figure 4:**
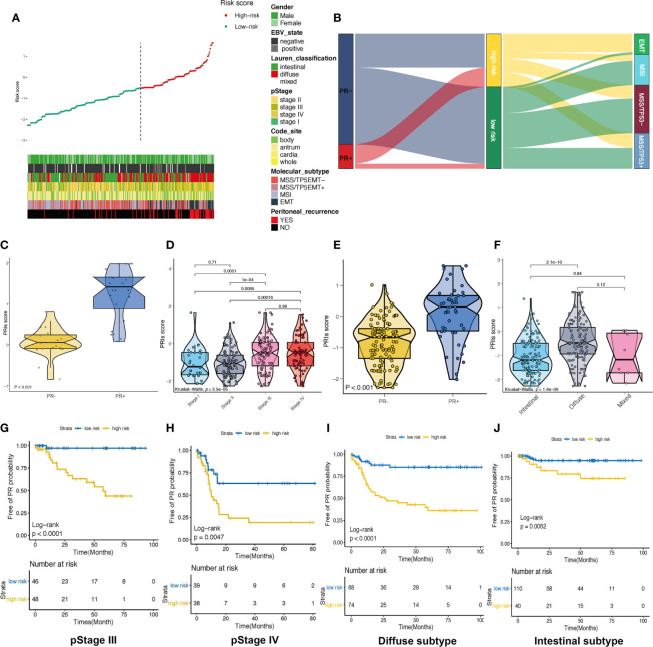
Association between PRIs and clinicopathological parameters **(A)** Summarizing the distribution of PRIs, and clinical characteristics. **(B)** Alluvial diagram of PR status in groups with different ACRG subtypes and PRIs. **(C)** Box diagram of PRIs in PR+ and PR- crowd in patients with EMT molecular subtypes. **(D)** Box diagram of PRIs with different pStage groups. **(E)** Box diagram for the differences in PRIs among PR+ and PR- people in pStage III and IV. **(F)** Differences in PRIs among PR+ and PR- people with different lauren types. **(G)** KM-curve for patients with high and low PRIs in stage III. **(H)** KM-curve for patients with high and low PRIs in stage IV. **(I)** KM-curve for patients with high and low PRIs in diffuse subtype. **(J)** KM-curve for patients with high and low PRIs in intestinal subtype.

It has been reported that the specific clinical factors, including tumor size, histopathology of biopsy sample, and tumor morphology, were significantly correlated with peritoneal relapse. CA19-9, lymphocyte count and NLR were also predictive factors for peritoneal relapse (26), we further explored the association between PRIs and valuable clinicopathological parameters (**Figure 4A**). As we can see, most of the patients with EMT molecular subtype, diffuse type of Lauren classification or pSTAGE in stage III to IV were considered to be in the high PRIs group and these patients occurred PR more frequently. In the Alluvial diagram, we can more intuitively see that most EMT group patients are considered to be in high-risk group of PR (**Figure 4B** and **Supplement Figure 1D**). Moreover, patients possessed PR+ have higher PRIs in patients with EMT molecular subtype (**Figure 4C**). So more attention should be paid to patients with EMT molecular subtypes, especially these with high PRIs. Similarly, patients with pStage III, IV had a much higher proportion patients proportion that were considered to be at high risk (**Figure 4D** and **Supplement Figure 1E**). Further analysis of patients with pstage III and IV showed that patients possessed PR+ had higher PRIs (**Figure 4E**). The Log-rank text and Kaplan-Meier curves showed within pstage III (**Figure 4G**) and pstage IV (**Figure 4H**) patients, patients with high PRIs did occur more peritoneal recurrence. Patients with diffused subtype possessed higher PRIs than intestinal subtype (**Figure 4F** and **Supplement Figure 1F**). Within patients with diffused type (**Figure 4I**) and intestinal type (**Figure 4J**), peritoneal relapse did occur more frequently in patients with high PRIs. Overall, PRIs showed general applicability in different subgroups.

### Pathway Enrichment Analysis Based on PRI

**Figure 5:**
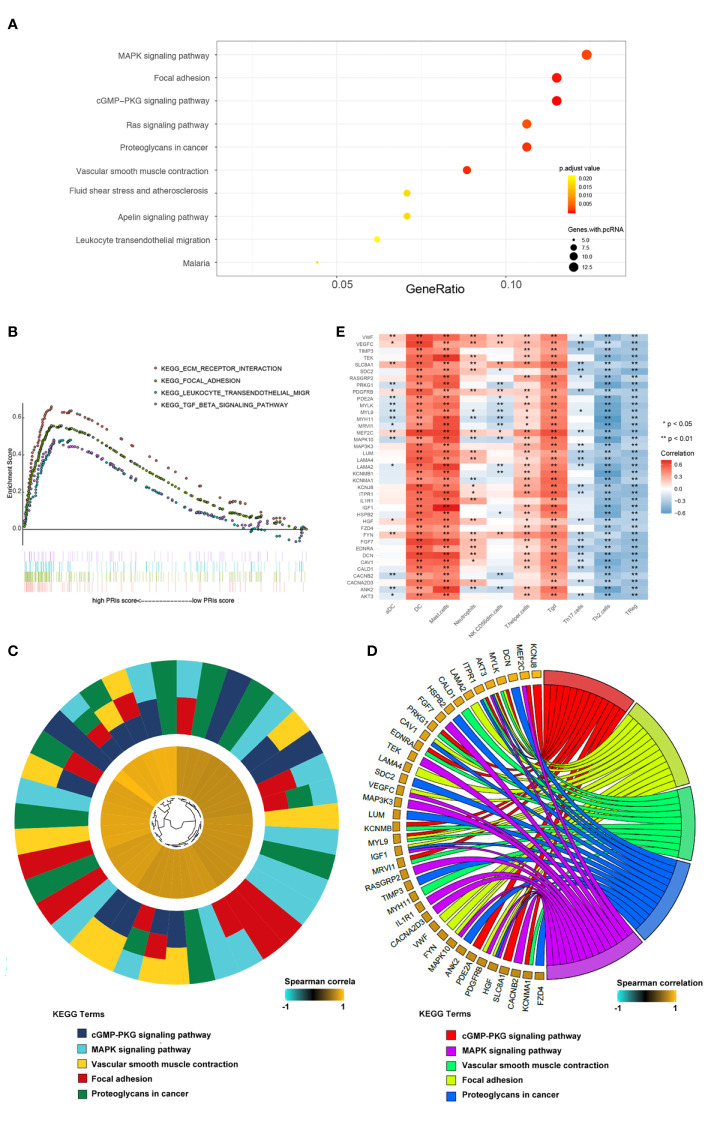
Pathway enrichment analysis based on PRIs. **(A)** Genes with spearman correlation for PRIs greater than 0.5 were used for KEGG analysis. These genes enriched in KEGG pathways “MAPK signaling pathway,” “Focal adhesion,” “cGMP-PKG signaling pathway,” and “Fas signaling pathway,” etc. Fold enrichment of each KEGG term is indicated by the x-axis and bar color. **(B)** GSEA terms that are significantly enriched in GSE62254 cohort. “KEGG_ECM_RECEPTOR_INTERACTION”,”KEGG_FOCAL_ADHESION”, “KEGG_LEUKOCYTE_TRANSENDOTHELIAL_MIGRATION”, “KEGG_MISMATCH_REPAIR”,”KEGG_NOTCH_SIGNALING_PATHWAY,” and “KEGG_TGB_BETA_SIGNALING_PATHWAY” was significantly enriched. **(C)** Hierarchical clustering of gene expression profiles of each KEGG pathways. **(D)** Chord plots show the relationship between genes and the KEGG pathways. **(E)** Correlation between the PR-related immune cells and genes in the KEGG pathways. *P < 0.05, **P < 0.01.

To elucidate the different biological characteristics between high risk and low risk patients, based on PRIs, we identified 214 genes with a spearman correlation with PRIs greater than 0.5 (**Table S4**), Further, the KEGG enrichment analysis, which was conducted by a cluster profile in R software, showed that the 214 genes were significantly enriched in MAPK signaling pathway, focal adhesion, cGMP-PKG signaling pathway, etc. (**Figure 5A**). In addition, visualized specifically enriched genes were presented in each KEGG term (**Figures 5C, D**). Next, we divided the patients into high and low groups according to PRIs for GSEA analysis, Focal adhesion and leukocyte transendothelial migration pathways were also enriched (**Figure 5B**). These results may demonstrate that focal adhesion and leukocyte transendothelial migration play a vital role in the occurrence of PR of GC. In order to explore whether immune cells can affect the activation of PR-related pathways, we further analyzed the relationship between the 10 PR-related immune cells and the 39 genes in the PR-related pathways obtained from **Figure 5D**. As shown in **Figure 5E**, DC, Mast cell, Tgd and T helper cell possessed strong positive correlation with most genes, while NK.CD56dim cells, Tregs, Th2 cells possessed strong negative correlation with these genes. This indicates that in the microenvironment of gastric cancer tumors, immune cells may regulate PR of gastric cancer by affecting the expression of these genes.

### Development and Validation of PRIs-Related Nomogram

**Figure 6:**
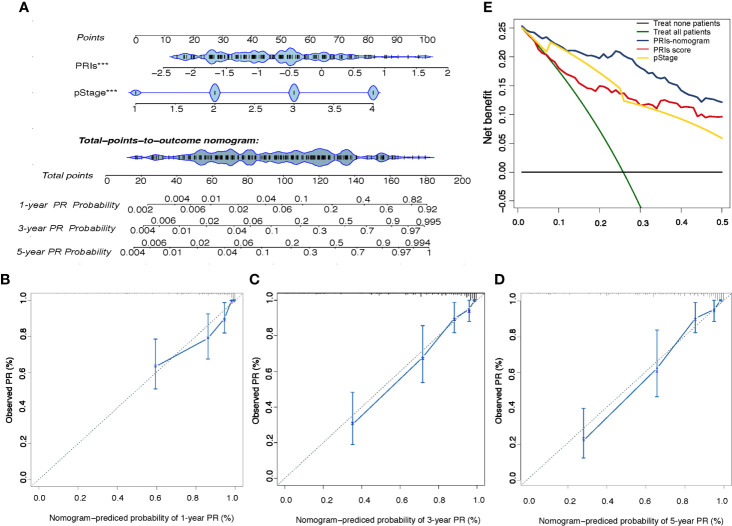
Development and Validation of the PRIs-related Nomogram **(A)** The peritoneal relapse related nomogram based on two predictors include PRIs and pStage. Each factor corresponds to its own score, and each score is added to obtain a total score. The total scores in 1-year, 3-year, 5-year PR probability represent the peritoneal relapse possibility within 1-year, 3-year and 5-year. **(B–D)** Calibration curve at the year of 1,3,5. The calibration curve describes the calibration of the fitting model according to the consistency between the predicted peritoneal relapse risk and the actual observations. The X axis represents the predicted peritoneal relapse risk, and the y axis represents the actual peritoneal relapse rate. Solid blue lines indicate the performance of nomogram. **(E)** Decision curve analysis for the PRIs-related nomogram. The Y axis measures net benefit. The blue line, yellow line, red line represent the PRIs-nomogram, pStage and PRIs separately, green line represents the assumption that all patients occurred peritoneal relapse, and the black line at the bottom represents the assumption that no patient occurred peritoneal relapse. The proportion of all false positive patients was subtracted from the proportion of true positive patients, and the net benefit was calculated by weighting the relative harm of abandoning treatment and the negative consequences of unnecessary treatment. Relative damage is calculated in terms of P_t_/(1-P_t_). P_t_ means that the expected benefit of treatment is equal to the expected benefit of avoiding treatment, at this point, the patient will choose treatment. The decision curve shows that even if the threshold probability of the patient or doctor is really small, using the PRIs nomogram in this study to predict peritoneal relapse brings more benefits than other methods.

In order to provide patients with more accurate prediction of PR, we included PRIs and clinical parameters with predictive value for PR in univariate analysis into multivariate analysis. The results showed that both in the internal training cohort and the internal validation cohort, only PRIs and pStage have stable predictive effect (**Table 1** and **Table S5**). Taking the principle of simple for patient measurement into account, our subsequent studies had excluded other clinical parameters. Based on PRIs and pStage, we constructed a PR nomogram (**Figure 6A**). The calibration curve shows that nomogram has stable predictive value at the time point of 1 year, 3 years and 5 years (**Figures 6B–D**). The DCA curve showed that combining PRIs and pStage can better provide medical decisions for patients (**Figure 6E**).

**Table 1:** Univariate and multivariate Cox analysis among PRIs and clinical features in training cohort.

Variables	Univariate analysis			Multivariate analysis		
	HR	95%CI	*P* value	HR	95%CI	*P* value
PRIs	3.230	2.124-4.910	<0.001	2.875	1.845-4.478	<0.001
Age(years)(<60 vs. ≥60)	1.027	0.470-2.244	0.948			
Gender(Male vs. Female)	0.780	0.358-1.699	0.532			
EBV	2.535	0.753-8.398	0.134			
Lauren	2.456	1.362-4.429	0.003			
pStage	4.156	2.286-7.556	<0.001	4.468	2.266-8.811	<0.001
Molecular subtype	1.385	0.985-1.948	0.061			

### Potential of PRIs as an Indicator of Immunotherapy Response in Patients With GC

Previous studies have shown that immune checkpoint inhibitory (ICI) genes and immunomodulatory genes can regulate immune infiltration. We further compared the expression patterns of ICI genes (TIM-3, PD-L1 and CTLA-4) and immunomodulatory gene (IL6, IL10, TGFB1) in patients with different PRIs stratification to reveal the complex crosstalk. Compared with patients with low PRIs, patients with high PRIs tend to express high ICI genes and immunomodulatory genes (**Supplement** **Figure 1G**). This trend further proves that the higher expression of ICI genes and immunomodulatory genes may be associated with frequent peritoneal recurrence in patients with gastric cancer and PRIs can serve as an indicator of immunotherapy response.

## Discussion

The presence of PR is related to poor prognosis in patients with GC. Although conventional imaging techniques have been applied to assess the metastasis, variation of sensitivity and specificity leads to limitation. Computed tomography (CT) is currently the primary imaging modality with a relatively low sensitivity for peritoneal metastasis of GC (27). Endoscopic ultrasonography (EUS) is invasive, highly operator-dependent and has a low detection rate of distant metastasis (28). The continuous technical improvements have shown that it can enhance high contrast resolution and characteristic soft-tissue of magnetic resonance imaging (MRI), especially the diagnostic value of small liver metastases (≤10mm) and peritoneal implantation (29–31). However, MRI is usually associated with higher costs, longer acquisition time and a lower robustness with no major oncology guidelines to recommend preoperative evaluation of GC (32–35). In contrast, the predictive value of 18F FDG PET/CT was high in several metastasis from GC including peritoneal metastasis, however, its limited usability and high costs would only make PET an alternative imaging modality (36). Besides, the peritoneal metastasis associated with serum biomarker, including CEA, CA125, CA199 and CA724 could only suggest the potential of metastasis with low specificity (37). Overall, a predictive approach or model for the PR risk after curative surgery of GC is urgently needed, making decision for the convenient scheme and dosage for GC patients with high risk of PR.

Multiple types of immune cells in TME promote tumor metastasis, either because they establish an immunosuppressive microenvironment within primary lesions or because they contribute to conditioning the pre-metastatic niche (38). These include Treg cells, myeloid-derived suppressor cells (MDSCs) (39), conventional CD11b+Ly6G+ neutrophils (40, 41) as well as macrophages (42). Instead, CD8+ T cells could directly suppress peritoneal metastasis by secreting cytokines including IFN-γ and GZMB (43–45), and Th1 CD4+ T cells T cells indirectly suppress metastasis by preventing vessel normalization (46). While Ly6G-neutrophils (47) and NK cells (48, 49) could develop innate anti-metastatic effects. Considering that recurrence and metastasis are the key factors affecting OS of patients with GC, several predictive models based on tumor- infiltration immune cells scores have been established to quantify the immune contexture and provide a statistical parameter for the prognosis of GC patients (16, 18, 19, 50). However, insufficient attention has been paid to the direct prediction of highly malignant event of PR for patients with GC.

Then, we established the PR risk immune predictive model (PRIs) of GC using LASSO-Cox regression algorithm (24). To the best of our knowledge, this study was the first bioinformatics model to predict the risk of PR for GC patients after surgical surgery. The final formula of the PRIs was composed of 10 types of immune cells including Th2 cells, mast cells, DCs, NK.CD56dim cells, Tregs, Tgd, Th17 cells, Neutrophils, αDCs and T helper cells. Similar to the previous research, CD56dim NK cells and neutrophils could directly exert the function of preventing PR (47–49, 51, 52). Besides, αDCs, as the important cells processing and presenting antigens to T cells (53), could elevate IL-1β and decreased IL-10 production to affect the metastatic ability of GC cells further (54–56). Zhao et al. reported a positive feedback that effects through TGFβ1 and IL-9 allowing cross-talk between Tregs and mast cells recently (57). The cross-talk suggests the underlying mechanism of nodes metastasis mediated by mast cells, which is in line with the findings of a previous study and ours (58). However, some inconsistency inevitably exists in this study, such as the suppressive roles of Tregs, Th2 and Th17 cells on PR (53, 59–61). The possible explanation is that CD4+ T helper cells are complex and heterogeneous at different developmental stages in GC (62). In the immune response, different subsets of CD4+ T helper cells communicate with other immune cells in TME to regulate the metastasis of GC, so the dynamic process and procedure may have effect on the multiple roles of T helper cells. Hence, to some extent, the integrated analysis of TME may better elucidate comprehensive interactions between the clinical characteristics of GC and infiltrating immune cells. With the help of ssGSEA algorithms, the results showed the PR risk of patients with high PRIs was higher than that of patients with low PRIs. Importantly, multivariate analysis demonstrated that PRIs was an independent predictor for PR, which was also verified by the nomogram integrated of PRIs, pStage and other clinical parameters. These results not only effectively certificated the efficacy and accuracy of our proposed immune predictive model, but also revealed the potential biological relationship between PR and immune infiltration landscape in GC.

We also uncovered a significant difference regarding the PRIs value in GC molecular subtypes and Lauren classification, with a higher value in the EMT subtype and diffuse type than other subtypes. Interestingly, it was also found that most patients with EMT subtype were allocated to the high risk subgroup with a higher percentage of relapse to peritoneum. Besides, the PRIs value was significantly higher in the III-IV pstage GC patients, and the PR subgroup in the III-IV pstage patients indeed had a higher PRIs. In addition, the Kaplan-Meier survival analysis showed a significantly better PR-free survival curve for patients with III stage and non-diffuse type than those with IV stage and diffuse type. Therefore, the results of biological function and clinical significance based on our PRIs value were basically consistent with the expert consensus (37). suggesting that our methodology to evaluate the risk of PR is a more predictive biomarker of gastric cancer.

Since the high-throughput sequence technology has been well improved currently, we further investigated the gene and pathway enrichment for in-depth understanding the underlying mechanism of our results. In previous researches, a large number of studies have elucidated the MAPK signaling, focal adhesion pathway, cGMP-PKG signaling pathway and etc. were closely or partially related to peritoneal metastasis of GC (63–67). Similar to the findings, the present study observed that 214 genes positively associated with PRIs enriched mainly in MAPK and focal adhesion pathway (**Figure 5A**), suggesting the major role of these two pathways taking on the peritoneal relapse of GC. Consistent with the results of our team before, several key molecules responsible for focal adhesion, including the family of integrin receptors and caveolin-1 (CAV-1) (68–70), involved in the peritoneal metastasis of GC, which were also shown in **Figure 5D**. Furthermore, by applying GSEA pathway enrichment, we also demonstrated the focal adhesion pathway, ECM receptor interaction, leukocyte transendothelial migration and TGF-β signaling pathway enriched almost in the patients with high PRIs (**Figure 5B**). In line with our findings, a previous research has indicated that peritoneal fibrosis induced by TGF-β provide a favorable environment for the dissemination of gastric cancer cells (67). In addition, the impaired cell-cell adhesion and enhanced cell-extracellular matrix (ECM) adhesion due to ECM remodeling and accumulation, has been recognized as an advancing factor of GC metastasis (71). This resource may offer mechanistic insights into the peritoneal metastasis of GC, suggesting the complex process is not only related to alteration of adhesive protein of tumors, but is also associated with microenvironment around the malignant tumor cells.

In particularly, patients with higher PRIs have higher expression levels of ICI genes (TIM-3, PD-L1 and CTLA-4). However, it is still controversial that PD-L1 expression is a favorable or adverse prognostic factor in GC according to previous researches (72–77). As illustrated before, TGF-β, IL-6, IL-10 and TIM3 could directly or indirectly promote EMT or peritoneal metastasis which was in line with our findings (78, 79), and it is reasonable to speculate that antibodies targeting these molecules may be a preferable choice for PR high risk subgroup. In fact, the experimental results have indicated the effect of anti-TIM-3 on the TME in variety tumors. A recent study illustrated the percentage of IFN-γ producing CD8+ T cells increased following anti-Tim-3 treatment to MC38 tumor bearing C57B6 mice, suggesting the enhancement of CD8+ T cell function in TME (80). Moreover, extensive data in preclinical tumor models and on-going I/II phase clinical trials in multiple tumors have shown the restoration of anti-tumor immunity by targeting Tim-3 (81), especially in combination with PD-1 blockade (82–84). Hence, based on our analysis about expression of immune related genes, emerging role of ICIs on application in the patients with GC, especially for the ones with high PR risk, are warranted to explore in the future.

Nevertheless, there are a few limitations that should be acknowledged. First, the study was based on publicly available datasets, and it was not possible to obtain the complete clinicopathologic parameters. This indicates the possibility that some patients with acute infection or immune system disorders, were inevitably included in this study. Ideally, such patients should have been excluded. Second, the public datasets in this study were based on two different platforms, though the RMA express were normalized, caution should be exerted when applying the conclusion of this study to samples tested using platforms other than GPL96 or GPL570. Finally, as all patients in the study were collected retrospectively, the potential bias due to unbalanced clinic characteristics with treatment heterogeneity could not be ignored. Thus, the results of our study should be validated in a prospective cohort of patients further.

In conclusion, this study illustrates the utility of immune cells infiltration in the prediction of PR of gastric cancer after surgery. The proposed PRIs model might provide more clinical information for shedding light on the improvement of personalized management of GC patients.

## Data Availability Statement

The datasets presented in this study can be found in online repositories. The names of the repository/repositories and accession number(s) can be found in the article/**Supplementary Material**.

## Author Contributions

CZ designed research and analyzed the data. DL provided clinical relevant opinions and wrote the discussion part. CL and YF Proofread the article. RY and YL helped collect data. YS and XC helped modified the discussion part. XQ supervised all works. All authors contributed to the article and approved the submitted version.

## Funding

This work was mainly supported by National Natural Science Foundation of China (NO.82003296, 81972751, 81602098, 81972331);Natural Science Foundation of Liaoning Province (2020-bs-040); Technological Special Project of Liaoning Province of China (2019020176-JH1/103); Science and Technology Plan Project of Liaoning Province (NO.2013225585); The General Projects of Liaoning Province Colleges and Universities (LFWK201706).

## Conflict of Interest

The authors declare that the research was conducted in the absence of any commercial or financial relationships that could be construed as a potential conflict of interest.

## References

[1] Bray F Ferlay J Soerjomataram I Siegel RL Torre LA Jemal A. Global Cancer Statistics 2018: GLOBOCAN Estimates of Incidence and Mortality Worldwide for 36 Cancers in 185 Countries. Ca-Cancer J Clin (2018) 68(6):394–424. 10.3322/caac.21492 30207593

[2] Yoo CH Noh SH Shin DW Choi SH Min JS. Recurrence Following Curative Resection for Gastric Carcinoma. Br J Surg (2000) 87(2):236–42. 10.1046/j.1365-2168.2000.01360.x 10671934

[3] Abe S Yoshimura H Tabara H Tachibana M Monden N Nakamura T. Curative Resection of Gastric Cancer: Limitation of Peritoneal Lavage Cytology in Predicting the Outcome. J Surg Oncol (1995) 59(4):226–9. 10.1002/jso.2930590405 7630168

[4] Bonnot PE Piessen G Kepenekian V Decullier E Pocard M Meunier B. Cytoreductive Surgery With or Without Hyperthermic Intraperitoneal Chemotherapy for Gastric Cancer With Peritoneal Metastases (Cyto-CHIP Study): A Propensity Score Analysis. J Clin Oncol (2019) 37(23):2028–40. 10.1200/JCO.18.01688 31084544

[5] Yang XJ Huang CQ Suo T Mei LJ Yang GL Cheng FL. Cytoreductive Surgery and Hyperthermic Intraperitoneal Chemotherapy Improves Survival of Patients With Peritoneal Carcinomatosis From Gastric Cancer: Final Results of a Phase III Randomized Clinical Trial. Ann Surg Oncol (2011) 18(6):1575–81. 10.1245/s10434-011-1631-5 PMC3087875 21431408

[6] Glehen O Gilly FN Arvieux C Cotte E Boutitie F Mansvelt B. Peritoneal Carcinomatosis From Gastric Cancer: A Multi-Institutional Study of 159 Patients Treated by Cytoreductive Surgery Combined With Perioperative Intraperitoneal Chemotherapy. Ann Surg Oncol (2010) 17(9):2370–7. 10.1245/s10434-010-1039-7 20336386

[7] Gentles AJ Newman AM Liu CL Bratman SV Feng W Kim D. The Prognostic Landscape of Genes and Infiltrating Immune Cells Across Human Cancers. Nat Med (2015) 21(8):938–45. 10.1038/nm.3909 PMC4852857 26193342

[8] Fridman WH Pages F Sautes-Fridman C Galon J. The Immune Contexture in Human Tumours: Impact on Clinical Outcome. Nat Rev Cancer (2012) 12(4):298–306. 10.1038/nrc3245 22419253

[9] Whiteside TL. The Tumor Microenvironment and its Role in Promoting Tumor Growth. Oncogene (2008) 27(45):5904–12. 10.1038/onc.2008.271 PMC3689267 18836471

[10] Schreiber RD Old LJ Smyth MJ. Cancer Immunoediting: Integrating Immunity’s Roles in Cancer Suppression and Promotion. Sci (New York NY) (2011) 331(6024):1565–70. 10.1126/science.1203486 21436444

[11] Grenader T Waddell T Peckitt C Oates J Starling N Cunningham D. Prognostic Value of Neutrophil-to-Lymphocyte Ratio in Advanced Oesophago-Gastric Cancer: Exploratory Analysis of the REAL-2 Trial. Ann Oncol (2016) 27(4):687–92. 10.1093/annonc/mdw012 26787231

[12] Mori M Shuto K Kosugi C Narushima K Hayashi H Matsubara H. An Increase in the Neutrophil-to-Lymphocyte Ratio During Adjuvant Chemotherapy Indicates a Poor Prognosis in Patients With Stage II or III Gastric Cancer. BMC Cancer (2018) 18(1):1261. 10.1186/s12885-018-5171-2 30558575 PMC6296109

[13] Hennequin A Derangere V Boidot R Apetoh L Vincent J Orry D. Tumor Infiltration by Tbet+ Effector T Cells and CD20+ B Cells is Associated With Survival in Gastric Cancer Patients. Oncoimmunology (2016) 5(2):e1054598. 10.1080/2162402X.2015.1054598 27057426 PMC4801425

[14] Lee HE Chae SW Lee YJ Kim MA Lee HS Lee BL. Prognostic Implications of Type and Density of Tumour-Infiltrating Lymphocytes in Gastric Cancer. Br J Cancer (2008) 99(10):1704–11. 10.1038/sj.bjc.6604738 PMC2584941 18941457

[15] Zhang R Li F Li H Yu J Ren X. The Clinical Significance of Memory T Cells and its Subsets in Gastric Cancer. Clin Transl Oncol (2014) 16(3):257–65. 10.1007/s12094-013-1066-5 23793812

[16] Zhang WJ Zhou ZH Guo M Yang LQ Xu YY Pang TH. High Infiltration of Polarized Cd163(+) Tumor-Associated Macrophages Correlates With Aberrant Expressions of CSCs Markers, and Predicts Prognosis in Patients With Recurrent Gastric Cancer. J Cancer (2017) 8(3):363–70. 10.7150/jca.16730 PMC5332886 28261336

[17] Song H Wang T Tian L Bai S Chen L Zuo Y. Macrophages on the Peritoneum are Involved in Gastric Cancer Peritoneal Metastasis. J Cancer (2019) 10(22):5377–87. 10.7150/jca.31787 PMC6775704 31632482

[18] Zeng D Li M Zhou R Zhang J Sun H Shi M. Tumor Microenvironment Characterization in Gastric Cancer Identifies Prognostic and Immunotherapeutically Relevant Gene Signatures. Cancer Immunol Res (2019) 7(5):737–50. 10.1158/2326-6066.CIR-18-0436 30842092

[19] Zeng D Zhou R Yu Y Luo Y Zhang J Sun H. Gene Expression Profiles for a Prognostic Immunoscore in Gastric Cancer. Br J Surg (2018) 105(10):1338–48. 10.1002/bjs.10871 PMC6099214 29691839

[20] Cristescu R Lee J Nebozhyn M Kim KM Ting JC Wong SS. Molecular Analysis of Gastric Cancer Identifies Subtypes Associated With Distinct Clinical Outcomes. Nat Med (2015) 21(5):449–56. 10.1038/nm.3850 25894828

[21] Subramanian A Tamayo P Mootha VK Mukherjee S Ebert BL Gillette MA. Gene Set Enrichment Analysis: A Knowledge-Based Approach for Interpreting Genome-Wide Expression Profiles. Proc Natl Acad Sci United States America (2005) 102(43):15545–50. 10.1073/pnas.0506580102 PMC1239896 16199517

[22] Bindea G Mlecnik B Tosolini M Kirilovsky A Waldner M Obenauf AC. Spatiotemporal Dynamics of Intratumoral Immune Cells Reveal the Immune Landscape in Human Cancer. Immunity (2013) 39(4):782–95. 10.1016/j.immuni.2013.10.003 24138885

[23] Hänzelmann S Castelo R Guinney J. GSVA: Gene Set Variation Analysis for Microarray and RNA-seq Data. BMC Bioinf (2013) 14:7. 10.1186/1471-2105-14-7 PMC3618321 23323831

[24] Tibshirani R. The Lasso Method for Variable Selection in the Cox Model. Stat Med (1997) 16(4):385–95. 10.1002/(sici)1097-0258(19970228)16:4<385::aid-sim380>3.0.co;2-3 9044528

[25] Yu G Wang LG Han Y He QY. clusterProfiler: An R Package for Comparing Biological Themes Among Gene Clusters. Omics J Integr Biol (2012) 16(5):284–7. 10.1089/omi.2011.0118 PMC3339379 22455463

[26] Ohi M Mori K Toiyama Y Mohri Y Okigami M Yasuda H. Preoperative Prediction of Peritoneal Metastasis in Gastric Cancer as an Indicator for Neoadjuvant Treatment. Anticancer Res (2015) 35(6):3511–8. 26026118

[27] Kwee RM Kwee TC. Modern Imaging Techniques for Preoperative Detection of Distant Metastases in Gastric Cancer. World J Gastroenterol (2015) 21(37):10502–9. 10.3748/wjg.v21.i37.10502 PMC4588073 26457011

[28] Mocellin S Pasquali S. Diagnostic Accuracy of Endoscopic Ultrasonography (EUS) for the Preoperative Locoregional Staging of Primary Gastric Cancer. Cochrane Database Syst Rev (2015) 2015(2):CD009944. 10.1002/14651858.CD009944.pub2 PMC6465120 25914908

[29] Maegerlein C Fingerle AA Souvatzoglou M Rummeny EJ Holzapfel K. Detection of Liver Metastases in Patients With Adenocarcinomas of the Gastrointestinal Tract: Comparison of (18)F-FDG PET/CT and MR Imaging. Abdom Imaging (2015) 40(5):1213–22. 10.1007/s00261-014-0283-x 25348732

[30] Soussan M Des Guetz G Barrau V Aflalo-Hazan V Pop G Mehanna Z. Comparison of FDG-PET/CT and MR With Diffusion-Weighted Imaging for Assessing Peritoneal Carcinomatosis From Gastrointestinal Malignancy. Eur Radiol (2012) 22(7):1479–87. 10.1007/s00330-012-2397-2 22358428

[31] Borggreve AS Goense L Brenkman HJF Mook S Meijer GJ Wessels FJ. Imaging Strategies in the Management of Gastric Cancer: Current Role and Future Potential of MRI. Br J Radiol (2019) 92(1097):20181044. 10.1259/bjr.20181044 30789792 PMC6580902

[32] Seevaratnam R Cardoso R McGregor C Lourenco L Mahar A Sutradhar R. How Useful is Preoperative Imaging for Tumor, Node, Metastasis (TNM) Staging of Gastric Cancer? A meta-analysis. Gastric Cancer (2012) 15 Suppl 1:S3–18. 10.1007/s10120-011-0069-6 21837458

[33] Renzulli M Clemente A Spinelli D Ierardi AM Marasco G Farina D. Gastric Cancer Staging: Is it Time for Magnetic Resonance Imaging? Cancers (Basel) (2020) 12(6):1402. 10.3390/cancers12061402 PMC7352169 32485933

[34] Wang FH Shen L Li J Zhou ZW Liang H Zhang XT. The Chinese Society of Clinical Oncology (CSCO): Clinical Guidelines for the Diagnosis and Treatment of Gastric Cancer. Cancer Commun (Lond) (2019) 39(1):10. 10.1186/s40880-019-0349-9 30885279 PMC6423835

[35] Smyth EC Verheij M Allum W Cunningham D Cervantes A Arnold D. Gastric Cancer: ESMO Clinical Practice Guidelines for Diagnosis, Treatment and Follow-Up. Ann Oncol (2016) 27(suppl 5):v38–49. 10.1093/annonc/mdw350 27664260

[36] Ozkan E Araz M Soydal C Kucuk ON. The Role of 18F-FDG-PET/CT in the Preoperative Staging and Posttherapy Follow Up of Gastric Cancer: Comparison With Spiral CT. World J Surg Oncol (2011) 9:75. 10.1186/1477-7819-9-75 21752303 PMC3148984

[37] 中国抗癌协会胃癌专业委员会. 胃癌腹膜转移防治中国专家共识. 中华胃肠外科杂志 (2017) 20(5):481–90. 10.3760/cma.j

[38] Lopez-Soto A Gonzalez S Smyth MJ Galluzzi L. Control of Metastasis by NK Cells. Cancer Cell (2017) 32(2):135–54. 10.1016/j.ccell.2017.06.009 28810142

[39] Vences-Catalan F Rajapaksa R Srivastava MK Marabelle A Kuo CC Levy R. Tetraspanin CD81 Promotes Tumor Growth and Metastasis by Modulating the Functions of T Regulatory and Myeloid-Derived Suppressor Cells. Cancer Res (2015) 75(21):4517–26. 10.1158/0008-5472.CAN-15-1021 26329536

[40] Liu Y Gu Y Han Y Zhang Q Jiang Z Zhang X. Tumor Exosomal Rnas Promote Lung Pre-metastatic Niche Formation by Activating Alveolar Epithelial TLR3 to Recruit Neutrophils. Cancer Cell (2016) 30(2):243–56. 10.1016/j.ccell.2016.06.021 27505671

[41] Wculek SK Malanchi I. Author Correction: Neutrophils Support Lung Colonization of Metastasis-Initiating Breast Cancer Cells. Nature (2019) 571(7763):E2. 10.1038/s41586-019-1328-7 31227820

[42] Georgoudaki AM Prokopec KE Boura VF Hellqvist E Sohn S Ostling J. Reprogramming Tumor-Associated Macrophages by Antibody Targeting Inhibits Cancer Progression and Metastasis. Cell Rep (2016) 15(9):2000–11. 10.1016/j.celrep.2016.04.084 27210762

[43] Senovilla L Vacchelli E Galon J Adjemian S Eggermont A Fridman WH. Trial Watch: Prognostic and Predictive Value of the Immune Infiltrate in Cancer. Oncoimmunology (2012) 1(8):1323–43. 10.4161/onci.22009 PMC3518505 23243596

[44] Ma Z Li W Yoshiya S Xu Y Hata M El-Darawish Y. Augmentation of Immune Checkpoint Cancer Immunotherapy With IL18. Clin Cancer Res (2016) 22(12):2969–80. 10.1158/1078-0432.CCR-15-1655 26755531

[45] Fujimori D Kinoshita J Yamaguchi T Nakamura Y Gunjigake K Ohama T. Established Fibrous Peritoneal Metastasis in an Immunocompetent Mouse Model Similar to Clinical Immune Microenvironment of Gastric Cancer. BMC Cancer (2020) 20(1):1014. 10.1186/s12885-020-07477-x 33081727 PMC7574408

[46] Tian L Goldstein A Wang H Ching Lo H Sun Kim I Welte T. Mutual Regulation of Tumour Vessel Normalization and Immunostimulatory Reprogramming. Nature (2017) 544(7649):250–4. 10.1038/nature21724 PMC5788037 28371798

[47] Hanna RN Cekic C Sag D Tacke R Thomas GD Nowyhed H. Patrolling Monocytes Control Tumor Metastasis to the Lung. Science (2015) 350(6263):985–90. 10.1126/science.aac9407 PMC4869713 26494174

[48] Sun JC Lanier LL. NK Cell Development, Homeostasis and Function: Parallels With CD8(+) T Cells. Nat Rev Immunol (2011) 11(10):645–57. 10.1038/nri3044 PMC4408539 21869816

[49] Cerwenka A Lanier LL. Natural Killer Cell Memory in Infection, Inflammation and Cancer. Nat Rev Immunol (2016) 16(2):112–23. 10.1038/nri.2015.9 26806484

[50] Jiang Y Zhang Q Hu Y Li T Yu J Zhao L. ImmunoScore Signature: A Prognostic and Predictive Tool in Gastric Cancer. Ann Surg (2018) 267(3):504–13. 10.1097/SLA.0000000000002116 28002059

[51] Saito H Osaki T Ikeguchi M. Decreased NKG2D Expression on NK Cells Correlates With Impaired NK Cell Function in Patients With Gastric Cancer. Gastric Cancer (2012) 15(1):27–33. 10.1007/s10120-011-0059-8 21626292

[52] Mao Z Zhang J Shi Y Li W Shi H Ji R. CXCL5 Promotes Gastric Cancer Metastasis by Inducing Epithelial-Mesenchymal Transition and Activating Neutrophils. Oncogenesis (2020) 9(7):63. 10.1038/s41389-020-00249-z 32632106 PMC7338464

[53] Subhash VV Yeo MS Tan WL Yong WP. Strategies and Advancements in Harnessing the Immune System for Gastric Cancer Immunotherapy. J Immunol Res (2015) 2015:308574. 10.1155/2015/308574 26579545 PMC4633567

[54] Rhee I Zhong MC Reizis B Cheong C Veillette A. Control of Dendritic Cell Migration, T Cell-Dependent Immunity, and Autoimmunity by Protein Tyrosine Phosphatase PTPN12 Expressed in Dendritic Cells. Mol Cell Biol (2014) 34(5):888–99. 10.1128/MCB.01369-13 PMC4023833 24366546

[55] Chang LL Wang SW Wu IC Yu FJ Su YC Chen YP. Impaired Dendritic Cell Maturation and IL-10 Production Following H. pylori stimulation in gastric cancer patients. Appl Microbiol Biotechnol (2012) 96(1):211–20. 10.1007/s00253-012-4034-z PMC3433674 22526791

[56] Kim JE Lee JY Kang MJ Jeong YJ Choi JA Oh SM. Withaferin A Inhibits Helicobacter Pylori-Induced Production of IL-1beta in Dendritic Cells by Regulating NF-Kappab and NLRP3 Inflammasome Activation. Immune Netw (2015) 15(6):269–77. 10.4110/in.2015.15.6.269 PMC4700403 26770181

[57] Zhao YB Yang SH Shen J Deng K Li Q Wang Y. Interaction Between Regulatory T Cells and Mast Cells Via IL-9 and TGF-beta Production. Oncol Lett (2020) 20(6):360. 10.3892/ol.2020.12224 33133260 PMC7590434

[58] Ammendola M Sacco R Zuccala V Luposella M Patruno R Gadaleta P. Mast Cells Density Positive to Tryptase Correlate With Microvascular Density in Both Primary Gastric Cancer Tissue and Loco-Regional Lymph Node Metastases From Patients That Have Undergone Radical Surgery. Int J Mol Sci (2016) 17(11):1905. 10.3390/ijms17111905 PMC5133903 27854307

[59] Su Z Sun Y Zhu H Liu Y Lin X Shen H. Th17 Cell Expansion in Gastric Cancer may Contribute to Cancer Development and Metastasis. Immunol Res (2014) 58(1):118–24. 10.1007/s12026-013-8483-y 24402773

[60] Lee K Hwang H Nam KT. Immune Response and the Tumor Microenvironment: How They Communicate to Regulate Gastric Cancer. Gut Liver (2014) 8(2):131–9. 10.5009/gnl.2014.8.2.131 PMC3964262 24672653

[61] Okita Y Ohira M Tanaka H Tokumoto M Go Y Sakurai K. Alteration of CD4 T Cell Subsets in Metastatic Lymph Nodes of Human Gastric Cancer. Oncol Rep (2015) 34(2):639–47. 10.3892/or.2015.4064 26081040

[62] Speiser DE Ho PC Verdeil G. Regulatory Circuits of T Cell Function in Cancer. Nat Rev Immunol (2016) 16(10):599–611. 10.1038/nri.2016.80 27526640

[63] Wu S Chen M Huang J Zhang F Lv Z Jia Y. Orai2 Promotes Gastric Cancer Tumorigenicity and Metastasis Through PI3K/Akt Signaling and MAPK-dependent Focal Adhesion Disassembly. Cancer Res (2020) 81(4):986–1000. 10.1158/0008-5472.Can-20-0049 33310726

[64] Dong H Liu H Zhou W Zhang F Li C Chen J. GLI1 Activation by non-Classical Pathway Integrin αβ/ERK1/2 Maintains Stem Cell-Like Phenotype of Multicellular Aggregates in Gastric Cancer Peritoneal Metastasis. Cell Death Dis (2019) 10: (8):574. 10.1038/s41419-019-1776-x 31366904 PMC6668446

[65] Ichikawa T Okugawa Y Toiyama Y Tanaka K Yin C Kitajima T. Clinical Significance and Biological Role of L1 Cell Adhesion Molecule in Gastric Cancer. Br J Cancer (2019) 121: (12):1058–68. 10.1038/s41416-019-0646-8 PMC6964673 31754264

[66] Zhang K Yang G Wu W Zhang J Xia X Jiang T. Decreased Expression of Caveolin-1 and E-Cadherin Correlates With the Clinicopathologic Features of Gastric Cancer and the EMT Process. Recent Pat Anticancer Drug Discov (2016) 11: (2):236–44. 10.2174/1574892811666160128151437 26817615

[67] Senbanjo L Chellaiah M Biology D. CD44: A Multifunctional Cell Surface Adhesion Receptor Is a Regulator of Progression and Metastasis of Cancer Cells. Front Cell Dev Biol (2017) 5:18. 10.3389/fcell.2017.00018 28326306 PMC5339222

[68] Wang X Che X Yu Y Cheng Y Bai M Yang Z. Hypoxia-Autophagy Axis Induces VEGFA by Peritoneal Mesothelial Cells to Promote Gastric Cancer Peritoneal Metastasis Through an Integrin α5-Fibronectin Pathway. J Exp Clin Cancer Res (2020) 39: (1):221. 10.1186/s13046-020-01703-x 33081836 PMC7576728

[69] Wang Y Song Y Che X Zhang L Wang Q Zhang X. Caveolin−1 Enhances RANKL−induced Gastric Cancer Cell Migration. Oncol Rep (2018) 40: (3):1287–96. 10.3892/or.2018.6550 PMC6072394 30015970

[70] Zang D Zhang C Li C Fan Y Li Z Hou K. LPPR4 Promotes Peritoneal Metastasis Via Sp1/integrin α/FAK Signaling in Gastric Cancer. Am J Cancer Res (2020) 10: (3):1026–44. 10.1200/jco.2016.34.4 PMC7136906 32266108

[71] Jang M Koh I Lee J Lim J Cheong J Kim P. Increased Extracellular Matrix Density Disrupts E-Cadherin/β-Catenin Complex in Gastric Cancer Cells. Biomater Sci (2018) 6: (10):2704–13. 10.1039/c8bm00843d 30151505

[72] Shigemori T Toiyama Y Okugawa Y Yamamoto A Yin C Narumi A. Soluble PD-L1 Expression in Circulation as a Predictive Marker for Recurrence and Prognosis in Gastric Cancer: Direct Comparison of the Clinical Burden Between Tissue and Serum Pd-L1 Expression. Ann Surg Oncol (2019) 26(3):876–83. 10.1245/s10434-018-07112-x 30565045

[73] Ito S Masuda T Noda M Hu Q Shimizu D Kuroda Y. Prognostic Significance of PD-1, Pd-L1 and CD8 Gene Expression Levels in Gastric Cancer. Oncology-Basel (2020) 98(7):501–11. 10.1159/000506075 32380498

[74] Xing X Guo J Ding G Li B Dong B Feng Q. Analysis of PD1, Pdl1, PDL2 Expression and T Cells Infiltration in 1014 Gastric Cancer Patients. Oncoimmunology (2018) 7(3):e1356144. 10.1080/2162402X.2017.1356144 29399387 PMC5790386

[75] Eto S Yoshikawa K Nishi M Higashijima J Tokunaga T Nakao T. Programmed Cell Death Protein 1 Expression is an Independent Prognostic Factor in Gastric Cancer After Curative Resection. Gastric Cancer (2016) 19(2):466–71. 10.1007/s10120-015-0519-7 26210691

[76] Thompson ED Zahurak M Murphy A Cornish T Cuka N Abdelfatah E. Patterns of PD-L1 Expression and CD8 T Cell Infiltration in Gastric Adenocarcinomas and Associated Immune Stroma. Gut (2017) 66(5):794–801. 10.1136/gutjnl-2015-310839 26801886 PMC4958028

[77] Wang Y Zhu C Song W Li J Zhao G Cao H. Pd-L1 Expression and CD8(+) T Cell Infiltration Predict a Favorable Prognosis in Advanced Gastric Cancer. J Immunol Res (2018) 2018:4180517. 10.1155/2018/4180517 30003113 PMC5996418

[78] Wu X Tao P Zhou Q Li J Yu Z Wang X. IL-6 Secreted by Cancer-Associated Fibroblasts Promotes Epithelial-Mesenchymal Transition and Metastasis of Gastric Cancer Via JAK2/STAT3 Signaling Pathway. Oncotarget (2017) 8(13):20741–50. 10.18632/oncotarget.15119 PMC5400541 28186964

[79] Wang R Song S Harada K Ghazanfari Amlashi F Badgwell B Pizzi MP. Multiplex Profiling of Peritoneal Metastases From Gastric Adenocarcinoma Identified Novel Targets and Molecular Subtypes That Predict Treatment Response. Gut (2020) 69(1):18–31. 10.1136/gutjnl-2018-318070 31171626 PMC6943252

[80] Yang M Du W Yi L Wu S He C Zhai W. Checkpoint Molecules Coordinately Restrain Hyperactivated Effector T Cells in the Tumor Microenvironment. Oncoimmunology (2020) 9(1):1708064. 10.1080/2162402x.2019.1708064 32076578 PMC6999836

[81] Acharya N Sabatos-Peyton C Anderson AC. Tim-3 Finds its Place in the Cancer Immunotherapy Landscape. J Immunother Cancer (2020) 8(1):e000911. 10.1136/jitc-2020-000911 32601081 PMC7326247

[82] Sakuishi K Apetoh L Sullivan JM Blazar BR Kuchroo VK Anderson AC. Targeting Tim-3 and PD-1 Pathways to Reverse T Cell Exhaustion and Restore Anti-Tumor Immunity. J Exp Med (2010) 207(10):2187–94. 10.1084/jem.20100643 PMC2947065 20819927

[83] Ngiow SF von Scheidt B Akiba H Yagita H Teng MW Smyth MJ. Anti-TIM3 Antibody Promotes T Cell IFN-γ-Mediated Antitumor Immunity and Suppresses Established Tumors. Cancer Res (2011) 71(10):3540–51. 10.1158/0008-5472.Can-11-0096 21430066

[84] Zhou Q Munger ME Veenstra RG Weigel BJ Hirashima M Munn DH. Coexpression of Tim-3 and PD-1 Identifies a CD8+ T-Cell Exhaustion Phenotype in Mice With Disseminated Acute Myelogenous Leukemia. Blood (2011) 117(17):4501–10. 10.1182/blood-2010-10-310425 PMC3099570 21385853

