# Supplementary figures and images for: Immune Landscape of Gastric Carcinoma Tumor Microenvironment Identifies a Peritoneal Relapse Relevant Immune Signature
Source: Front Immunol. 2021 May 13;12:651033. doi: 10.3389/fimmu.2021.651033 (PMC8155484; doi:10.3389/fimmu.2021.651033)

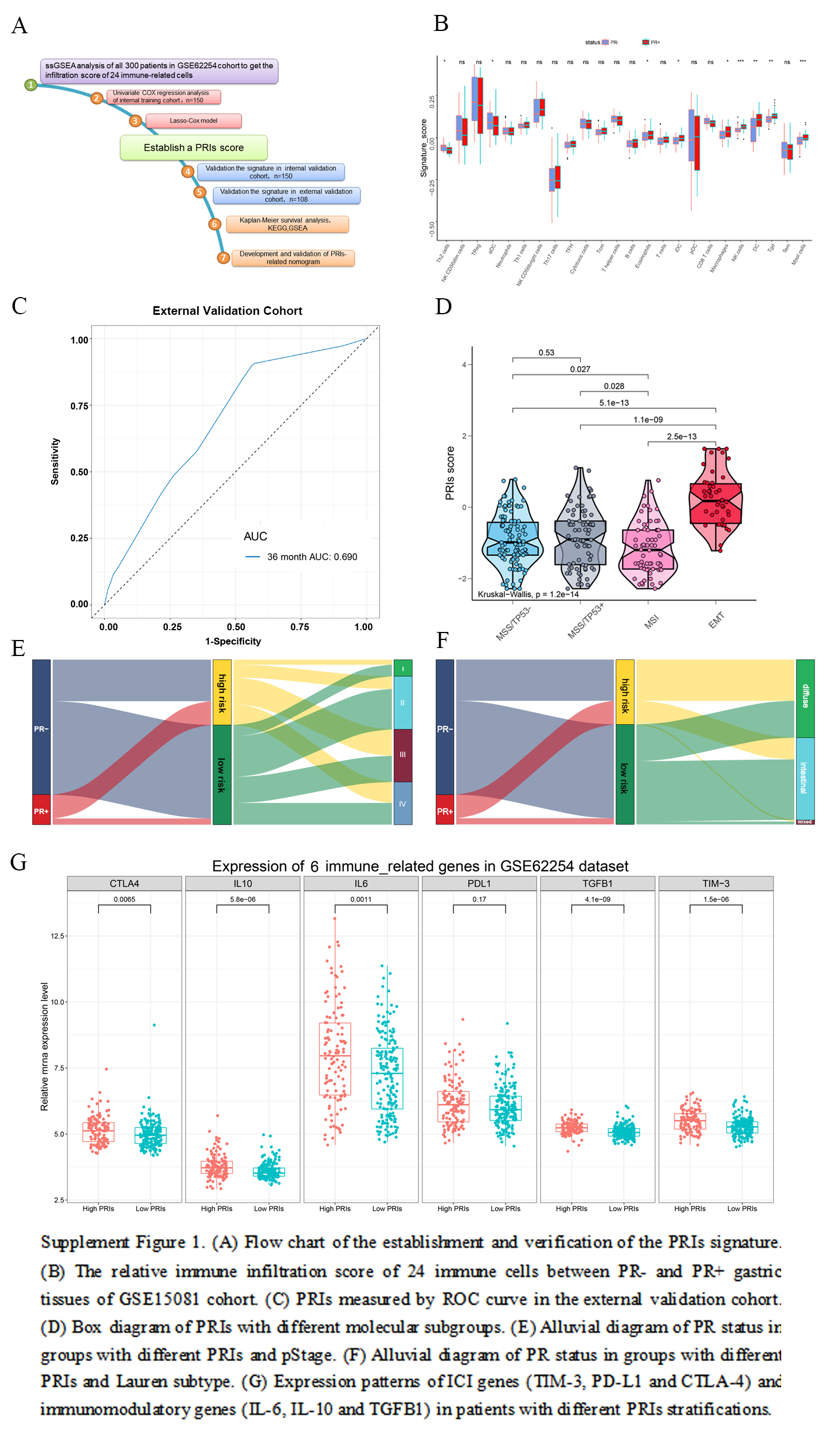

Supplement: Supplementary file 1 [file Image_1.tif]
