# Supplementary material for: Immune Landscape of Gastric Carcinoma Tumor Microenvironment Identifies a Peritoneal Relapse Relevant Immune Signature
Source: Front Immunol. 2021 May 13;12:651033. doi: 10.3389/fimmu.2021.651033 (PMC8155484; doi:10.3389/fimmu.2021.651033)
Supplement: Supplementary file 6 [file Table_5.docx]

| Variables | Univariate analysis | | |  | Multivariate analysis | | |
| --- | --- | --- | --- | --- | --- | --- | --- |
|  | HR | 95%CI | *P* value |  | HR | 95%CI | *P* value |
| PRIs | 3.321 | 2.261-4.879 | <0.001 |  | 2.230 | 1.526-3.259 | <0.001 |
| Age(years)(<60 vs. ≥60) | 0.635 | 0.279-1.445 | 0.280 |  |  |  |  |
| Gender(Male vs. Female) | 0.370 | 0.176-0.777 | 0.009 |  |  |  |  |
| EBV | 0.429 | 0.058-3.170 | 0.407 |  |  |  |  |
| Lauren | 1.778 | 0.991-3.190 | 0.054 |  |  |  |  |
| pStage | 3.857 | 2.299-6.471 | <0.001 |  | 2.948 | 1.667-5.213 | <0.001 |
| Molecular subtype | 1.705 | 1.178-2.469 | 0.005 |  |  |  |  |

**Supplement Table 5 Univariate and multivariate Cox analysis among PRIs and clinical features in validation cohort.**
